# Type 1 equilibrative nucleoside transporter regulates astrocyte-specific glial fibrillary acidic protein expression in the striatum

**DOI:** 10.1002/brb3.283

**Published:** 2014-09-19

**Authors:** David J Hinton, Moonnoh R Lee, Jin Sung Jang, Doo-Sup Choi

**Affiliations:** 1 Department of Psychiatry and Psychology, Mayo Clinic, College of MedicineRochester, Minnesota, 55905; 2 Department of Molecular Pharmacology and Experimental Therapeutics, Mayo Clinic, College of MedicineRochester, Minnesota, 55905; 3 Neurobiology of Disease Program, Mayo Clinic, College of MedicineRochester, Minnesota, 55905; 4 Department of Biochemistry and Molecular Biology, Mayo Clinic, College of MedicineRochester, Minnesota, 55905; 5 Division of Pulmonary and Critical Care Medicine, Mayo Clinic, College of MedicineRochester, Minnesota, 55905

**Keywords:** Adenosine transporter, astrocyte, ENT1, GFAP, microarray

## Abstract

**Background:**

Adenosine signaling has been implicated in several neurological and psychiatric disorders. Previously, we found that astrocytic excitatory amino acid transporter 2 (EAAT2) and aquaporin 4 (AQP4) are downregulated in the striatum of mice lacking type 1 equilibrative nucleoside transporter (ENT1).

**Methods:**

To further investigate the gene expression profile in the striatum, we preformed Illumina Mouse Whole Genome BeadChip microarray analysis of the caudate–putamen (CPu) and nucleus accumbens (NAc) in ENT1 null mice. Gene expression was validated by RT-PCR, Western blot, and immunofluorescence. Using transgenic mice expressing enhanced green fluorescence protein (EGFP) under the control of the glial fibrillary acidic protein (GFAP) promoter, we examined EGFP expression in an ENT1 null background.

**Results:**

Glial fibrillary acidic protein was identified as a top candidate gene that was reduced in ENT1 null mice compared to wild-type littermates. Furthermore, EGFP expression was significantly reduced in GFAP-EGFP transgenic mice in an ENT1 null background in both the CPu and NAc. Finally, pharmacological inhibition or siRNA knockdown of ENT1 in cultured astrocytes also reduced GFAP mRNA levels.

**Conclusions:**

Overall, our findings demonstrate that ENT1 regulates GFAP expression and possibly astrocyte function.

## Introduction

Type 1 equilibrative nucleoside transporter (ENT1) is ubiquitously expressed and is the primary transporter of adenosine (Young et al. 2008). Mice lacking ENT1 develop normally and have been used to study the importance of adenosine transport in cardioprotection, postischemic blood flow during kidney injury, uptake of PET tracers that accumulate in proliferating tissues, and bone disorders (Paproski et al. 2010; Rose et al. 2010, 2011; Grenz et al. 2012; Warraich et al. 2013; Hinton et al. 2014). In the central nervous system (CNS), ENT1 null mice have been used to investigate the contribution of adenosine to the fine-tuning of glutamatergic signaling with regards to addictive disorders (Choi et al. 2004; Nam et al. 2011, 2013; Hinton et al. 2012). Mice lacking ENT1 exhibit reduced excitatory amino acid transporter type 2 (EAAT2) and aquaporin type 4 (AQP4) but normal levels of glutamine synthetase (GS) (Wu et al. 2010, 2011; Lee et al. 2013), suggesting that ENT1 impacts astrocyte function.

Glial fibrillary acidic protein (GFAP) is known as a primary marker for astrocytes. GFAP has diverse cellular functions and is abundantly expressed in astrocytes of the CNS. However, transgenic animal models have demonstrated that GFAP is expressed in neurons, especially during development, as well as other cell types outside the CNS [reviewed in (Middeldorp and Hol 2011)]. Mice lacking GFAP present with astrocytes that lack GFAP intermediate filaments but develop otherwise normally (Pekny et al. 1995).

Here we investigated the effect of deletion of ENT1 on the expression of GFAP. We found that GFAP is reduced in the striatum of ENT1 null mice compared to wild-type mice. These data suggest that dampened ENT1 expression and thus adenosine homeostasis alters astrocyte function.

## Materials and Methods

### Animals

ENT1 null mice were generated as described (Choi et al. 2004). We used F2 generation hybrid mice with a C57BL/6J × 129X1/SvJ genetic background to minimize the risk of false positives or negatives in gene expression that could be influenced by a single-genetic background (Crusio et al. 2009). GFAP-EGFP mice were provided by Dr. Helmut Kettenmann (Max Delbrück Center for Molecular Medicine) (Nolte et al. 2001). We crossed GFAP-EGFP mice in a FVB/N background with ENT1 null mice in a C57BL/6J background, then crossed the GFAP-EGFP/ENT1^+/−^ with GFAP-EGFP/ENT1^+/−^ mice to generate GFAP-EGFP/ENT1^+/+^ or GFAP-EGFP/ENT1^−/−^ F2 mice with C57BL/6J × FVB/N genetic background. We used 8- to 16-week-old male littermates for all experiments. Mice were housed in standard Plexiglas cages with food and water ad libitum. The colony room was maintained on a 12-h light/12-h dark cycle with lights on at 6:00 a.m. Animal care and handling procedures were approved by the Mayo Clinic Institutional Animal Care and Use Committees in accordance with NIH guidelines.

### Illumina microarray

(1) *Total RNA isolation*. RNA was extracted using RNAeasy-Mini kit (Qiagen, Valencia, CA) (Wu et al. 2010, 2011; Lee et al. 2013). Quality and concentration of RNA were confirmed using Picochip (Agilent, Santa Clara, CA). (2) *Target (labeled cRNA) Preparation*. Total RNA (500 ng) was used to label cRNA. In the first step, single-stranded cDNA was synthesized by reverse transcription, which was converted into double stranded cDNA and purified using the Illumina TotalPrep RNA Amplification Kit (Life Technologies, Carlsbad, CA). An in vitro transcription (IVT) reaction was carried out overnight in the presence of biotinylated UTP and CTP to produce biotin-labeled cRNA from the double stranded cDNA. The cRNA from the IVT reaction was purified using the same Amplification Kit. (3) *Hybridization to the Chip*. After the quality control assessment, 1.5 *μ*g of cRNA was hybridized to the Illumina's MouseWG-6 v2.0 Expression BeadChips. The array was hybridized for 16 h in a hybridization oven with a rocking platform at 58°C. The array chip undertook a series of washes before it is stained with streptavidin-Cy3. After the staining, it was washed and dried. Then, the array was scanned using the Illumina BeadArray reader. (4) *Array data analysis*. The images were analyzed using the GenomeStudio software (Illumina, San Diego, CA) and data were analyzed according to Illumina's instructions. Both types of raw microarray data were processed and normalized through the GenomeStudio software, version 3.0, using the quantile normalization method. The normalized data were then log_2_ transformed and analyzed using the Partek Genomics Suite (Partek Inc., St. Louis, MO). Raw microarray data from the GenomeStudio software were exported. Analyses for both data types were performed on the log base 2 scale. For each data type, all samples were normalized together as study groups should not have wholesale changes in RNA concentration using appropriate model-based algorithms (Ballman et al. 2004; Oberg et al. 2008). Data were analyzed using linear models (ANOVA models) (Kerr et al. 2000; Wolfinger et al. 2001; Hill et al. 2008; Oberg et al. 2008; Oberg and Vitek 2009) together with empirical Bayes methods (Smyth 2004), which are appropriate for mitigating the risk of false discovery in small sample sizes. Contrast statements were used to assess the primary hypothesis of knockout versus wild type *via t*-tests. Data are expressed as mean fold change ± SEM. Volcano plots and per gene or protein dot plots were used to graphically display results. The false discovery rate (FDR), which is the expected proportion of false discoveries amongst the rejected hypotheses (Benjamini and Hochberg 1995), was set at 0.0001 for the CPu or 0.05 for the NAc to address possible issues with multiple comparisons. The quality control parameters used were (1) *housekeeping controls*-The intactness of the biological specimen was monitored by the housekeeping gene controls. These controls consisted of probes to housekeeping genes, two probes per gene, which should be expressed in any cellular sample. (2) *negative controls-*We used probes that do not match with any targets in the genomes. The GenomeStudio application used the signals and signal standard deviation to these probes to establish gene expression detection limits. (3) *hybridization controls*-three types of controls were used for hybridization, Cy3-labeled Hyb control, low stringency control, and high stringency control.

### Bioinformatic analysis

Genes identified from our microarray study were uploaded into Ingenuity Pathway Analysis (IPA) for stratification and categorization of direct and indirect network interactions using IPA's functional analysis algorithm and curated IPA Ingenuity Knowledge Base (IPAIKB). Two data sets of identified genes were entered, one corresponding to the CPu and the other to the NAc. A CORE comparative analysis for the two brain regions was performed using default IPA settings, except for assigning CNS only tissues and excluding cancer cell lines. To minimize the incidence of false positive results, expression value threshold filters were set to a 1.5 fold change ratio in the CPu and 1.25 fold change ratio in the NAc between wild-type and ENT1 null mice and a gene detection multiple testing corrected confidence value of *P *<* *0.0001 for the CPu and *P *<* *0.001 for the NAc. With these criteria, IPA was able to generate a reference data set consisting of all significant and nonsignificant genes identified in the CPu and NAc, as well as a focus gene data set consisting of differentially expressed genes derived from the reference data set. Biological functions, disease states, and canonical pathways associated with our data set were generated by IPA.

### Immunofluorescence

Mice were anesthetized with pentobarbital (80 mg/kg, *intraperitoneal injection*) and perfused *via* the aorta with 4% paraformaldehyde (Sigma-Aldrich, St. Louis, MO) in PBS. Brains were removed and postfixed for 24 h in the same fixative at 4°C. Brains were immersed in 30% sucrose for 24 h, frozen, and cut in 35 *μ*m sections using a cryostat (Leica, Germany). Free-floating sections were incubated in 50% alcohol for 20 min, followed by 10% normal donkey serum in PBS for 30 min, and then antibodies against GFAP (1:100; Cell Signaling, Danvers, MA) overnight. Sections were then incubated in 2% normal donkey serum in PBS for 10 min followed by Alexa 488-conjugated secondary goat anti-mouse antibody (1:1000; Cell Signaling, Danvers, MA) for 2 h. Images from each brain region of interest (CPu, NAc core, NAc shell) were obtained using an LSM 510 confocal laser scanning microscope (Carl Zeiss, Germany). Areas of GFAP and GFAP-EGFP-positive astrocytes within regions of interest (450 *μ*m × 450 *μ*m) were quantified using NIH Image J software (Bethesda, MD).

### Real-time RT-PCR

Mice were anesthetized with carbon dioxide and rapidly decapitated. CPu and NAc tissues were isolated under a surgical microscope. To measure mRNA levels, real-time quantitative RT-PCR was performed with the iCycler IQ Real-Time PCR detection system (Bio-Rad Laboratories, Inc., Hercules, CA) using QuantiTect SYBR Green RT-PCR Kit (Qiagen, Valencia, CA). Total RNA was isolated using RNAeasy-Mini kit (Qiagen) for analysis of gene expression levels using quantitative RT-PCR as described (Wu et al. 2010). Gene-specific primers for GFAP and GAPDH were purchased (Qiagen). The following real-time RT-PCR protocol was used for all genes: reverse transcription step for 30 min at 50°C, then denaturation at 95°C for 15 min to activate the HotStart enzyme, followed by an additional 45 cycles of amplification and quantification (15 sec at 94°C; 10 sec at 55°C; 30 sec at 72°C), each with a single fluorescence measurement. The mRNA expressions of GFAP were normalized by GAPDH as a housekeeping gene. Fold changes were calculated by subtracting GAPDH Ct values from Ct values for the gene of interest using the 2^−ΔΔCt^ method (Livak and Schmittgen 2001).

### Western blot

Mice were anesthetized with carbon dioxide and rapidly decapitated. Brains were quickly removed and dissected to isolate the CPu and NAc. Briefly, tissues were homogenized in a solution containing 50 mmol/L Tris buffer (pH 7.4), 2 mmol/L EDTA, 5 mmol/L EGTA, 0.1% SDS, protease inhibitor cocktail (Roche, Germany), and phosphatase inhibitor cocktail type I and II (Sigma-Aldrich, St. Louis, MO). Homogenates were centrifuged at 500 ***g*** at 4°C for 15 min and supernatants were collected. Proteins were analyzed using Bradford protein assay (BioRad, Hercules, CA). Proteins were separated by 4–12% NuPAGE™ Bis Tris gels at 130 V for 2 h, transferred onto PVDF membranes at 30 V for 1 h (Invitrogen, Carlsbad, CA), and analyzed using antibodies against GFAP (1:1000; Cell Signaling, Danvers, MA) and GAPDH (1:1000; Millipore, Billerica, MA). Blots were developed using chemiluminescent detection reagents (Pierce, Rockford, IL). Chemiluminescent bands were detected on a Kodak Image Station 4000R scanner (New Haven, CT) and quantified using NIH Image J software.

### Astrocyte culture

The astrocytic cell line, C8-D1A, was obtained from ATCC (American Type Culture Collection, Manassas, VA), which was cloned from the mouse cerebellum (Alliot and Pessac 1984). As we previously described (Wu et al. 2010), cells were maintained in Dulbecco's modified Eagle medium containing glucose (Invitrogen, Carlsbad, CA), 10% heat-inactivated fetal bovine serum (FBS; ATCC, American Type Culture Collection, VA), 1% L-glutamine (Gibco, Auckland, New Zealand), and 1% Antibiotic-Antimycotic (Invitrogen, Carlsbad, CA). Monolayers were cultured at 37°C in the presence of 5% CO_2_/95% O_2_ (normoxia) in a fully humidified atmosphere with medium replacement every 2–3 days.

### ENT1 inhibition and knockdown in the astrocytes

Nitrobenzylthioinosine (NBTI; Sigma-Aldrich), an ENT1-specific inhibitor, was used to examine the effect of the pharmacological inhibition of ENT1 on GFAP mRNA expression levels in a cerebellar (C8-D1A) astrocytic cell line. Cells were separated into three groups: control (DMSO incubation for 24 h), NBTI (10 *μ*mol/L NBTI incubation for 24 h), and NBTI+wash (10 *μ*mol/L NBTI incubation for 24 h followed by 24 h of incubation in new media). Following treatment, cells were harvested and mRNA levels were measured. We also examined the effect of ENT1 knockdown on GFAP mRNA expression levels in a cerebellar (C8-D1A) astrocytic cell line. The target sequence of Slc29a1-3 siRNA for mouse ENT1 is 5′-AAGATTGTGCTCATCAATTCA-3′. siRNAs for Slc29a1 or scrambled siRNA (30 nmol/L) were transfected into 5 × 10^5^ astrocytes in a 6-well plate using 4 *μ*L Lipofectamine 2000 with Plus reagent (Invitrogen, Carlsbad, CA). Twenty-four hours after the transfection, total RNA was isolated using RNAeasy-Mini kit (Qiagen) and the expression levels of GFAP and GAPDH mRNA were measured by real-time RT-PCR. We have previously shown that 24 h following ENT1 siRNA transfection is sufficient to regulate mRNA expression of other astrocytic genes including aquaporin-4 (AQP4) and excitatory amino acid transporter 2 (EAAT2) in astrocyte cell culture (Lee et al. 2013). Furthermore, in this same study, we utilized 24 h of NBTI exposure to downregulate AQP4 and EAAT2 in astrocyte cell culture. In addition, we have carried out time-dependent experiments on EAAT2 mRNA levels in astrocyte cell culture using and ENT1 inhibitor and have shown that even 3 h of exposure is sufficient to downregulate EAAT2 (Wu et al. 2010).

### Statistical analysis

All data were expressed as mean ± SEM (standard error mean) and were analyzed by unpaired two-tailed *t-*tests or one-way analysis of variance (ANOVA) followed by Tukey post hoc test. Results of comparisons were considered significantly different if the *P* value was <0.05.

## Results

### Microarray

Illumina's Mouse-WG6 v2.0 BeadChip format, which enabled us to interrogate more than 45,200 transcripts and to profile six samples simultaneously on a single chip (Fan et al. 2006), was used. As shown in Table 1, we identified 747 differentially expressed genes in the CPu of ENT1 null mice compared to wild-type littermates. A false discovery rate (FDR) of <0.0001, a *P* value (one-way ANOVA between genotypes) of <0.0001, and a fold change >1.5 were used as inclusion criteria for the CPu. In addition, 162 differentially expressed genes were identified in the NAc of ENT1 null mice compared to wild-type mice. An FDR <0.05, *P* value (one-way ANOVA between genotypes) <0.001, and a fold change >1.25 were used as inclusion criteria for the NAc.

**Table 1:** Summary of microarray data.

	CPu	NAc
No. of Mice	8 WT + 10 KO	6 WT + 6 KO
FDR	<0.0001	<0.05
*P* Value	<0.0001	<0.001
Fold Δ	>1.5	>1.25
No. genes	747	162

CPu, caudate-putamen; NAc, nucleus accumbens; FDR, false discovery rate; Fold Δ, KO/WT ratio.

### Ingenuity pathway analysis (IPA)

**Figure 1:**
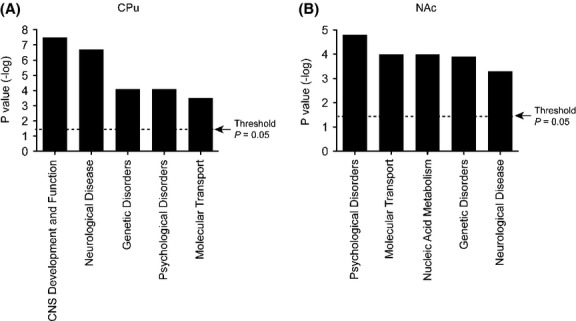
Top functional pathways identified by Ingenuity Pathway Analysis (IPA) based on significantly changed genes between ENT1 null and wild-type mice from microarray analysis. (A) CNS development and function, neurological disease, genetic disorders, psychological disorders, and molecular transport were identified as the top five functional pathways in the CPu that were above a threshold set at *P *=* *0.0001. (B) Psychological disorders, molecular transport, nucleic acid metabolism, genetic disorders, and neurological disease were identified as the top five functional pathways in the NAc that were above a threshold set at *P *=* *0.05.

In the CPu, Ingenuity Pathway Analysis (IPA) identified CNS development and function, neurological disease, genetic disorders, psychological disorders, and molecular transport as top functional pathways and in the NAc, psychological disorders, molecular transport, nucleic acid metabolism, genetic disorders, and neurological disease were identified as top functional pathways (Fig.1A and B). Based on these top functional pathways, we were highly interested in neurological disease and psychological disorders in the CPu and NAc. Since ENT1 null mice have been used as a model of excessive ethanol consumption (Choi et al. 2004; Nam et al. 2011, 2013; Hinton et al. 2012), several recent animal studies further illustrate that ENT1 gene expression is inversely correlated with ethanol drinking (Bell et al. 2009; Sharma et al. 2010) and, recent human genetic association studies demonstrate that variants of ENT1 are associated with an alcohol abuse phenotype in women (Gass et al. 2010) and alcoholics with a history of withdrawal seizures (Kim et al. 2011) we were mainly interested in genes that were altered specifically in the neurological disease and psychological disorders functional pathways. Several key genes in each of these two functional pathways that warrant further investigation were identified to be differentially expressed in ENT1 null mice compared to wild-type littermates in both the CPu (Tables 2 and 3) and NAc (Tables 4 and 5). A list of all significantly changed genes between ENT1 null and wild-type mice in the NAc and CPu are included in Supplemental Tables S1 and S2. Since we have previously identified several astrocytic genes to be altered in ENT1 null mice relative to wild-type littermates and have already functionally validated EAAT2 and AQP4 (Wu et al. 2010, 2011; Lee et al. 2013), we chose to pursue glial fibrillary acidic protein (GFAP) in the current manuscript.

**Table 2:** A list of differentially expressed genes of the neurological disease functional pathway in the CPu between ENT1 wild-type and null mice.

ID	Gene Name	Fold Δ	*P* Value
Atxn1	Ataxin 1	−2.71	1.62E-05
Aqp4	Aquaporin 4	−2.63	7.54E-06
Prkcbp1	Protein kinase C-binding protein 1 (*Zinc finger, MYND-type containing 8*)	−2.42	8.71E-07
Gfap	Glial fibrillary acidic protein	−2.05	2.82E-07
Slc5a7	Solute carrier family 5 (choline transporter), member 7	−2.04	1.41E-07
Gria1	Glutamate receptor, ionotropic, AMPA1 (alpha 1)	−1.97	2.49E-05
Prkce	Protein kinase C, epsilon	−1.92	2.38E-06
Scn3a	Sodium channel, voltage-gated, type III, alpha	−1.86	5.36E-05
Atp6v1a	ATPase, H+ transporting, lysosomal V1 subunit A	−1.83	3.51E-05
Ptprd	Protein tyrosine phosphatase, receptor type, D	−1.81	1.02E-08
Grin1	Glutamate receptor, ionotropic, NMDA1 (zeta 1)	−1.77	1.76E-06
Nrgn	Neurogranin	−1.75	2.82E-08
Slc1a4	Solute carrier family 1 (glutamate/neutral amino acid transporter), member 4	−1.71	4.00E-10
Syn1	Synapsin I	−1.68	7.95E-06
Adora1	Adenosine A1 receptor	−1.64	6.18E-08
Prkaca	Protein kinase, cAMP-dependent, catalytic, alpha	−1.62	4.03E-10
Gabrb2	Gamma-aminobutyric acid (GABA) A receptor, subunit beta 2	−1.56	5.79E-06
Atp2b2	ATPase, Ca++ transporting, plasma membrane 2	−1.54	3.21E-05
Comt	Catechol-O-methyltransferase 1	−1.53	1.50E-05
Kcnq2	Potassium voltage-gated channel, subfamily Q, member	−1.51	1.89E-07
Slc1a3	Solute carrier family 1 (glial high-affinity glutamate transporter), member 3	1.52	5.81E-08
Kcnc1	Potassium voltage-gated channel, Shaw-related subfamily, member 1	1.53	1.11E-05
Kcnab2	Potassium voltage-gated channel, shaker-related subfamily, beta member 2	1.73	5.79E-09

Fold Δ represents ENT1 null/wild-type ratio.

**Table 3:** A list of differentially expressed genes of the psychological disease functional pathway in the CPu between ENT1 wild-type and null mice.

ID	Gene Name	Fold Δ	*P* Value
Ccnd2	Cyclin D2	−2.97	2.14E-05
Nrxn3	Neurexin-3-alpha	−2.86	7.64E-06
Nell2	Neural Epidermal Growth Factor-Like 2	−2.76	2.28E-10
Zfhx3	Zinc Finger Homeobox 3	−2.76	3.56E-08
Atxn1	Ataxin 1	−2.71	1.62E-05
Aqp4	Aquaporin 4	−2.63	7.54E-06
Kalrn	Kalirin, RhoGEF Kinase	−2.58	7.81E-07
Susd4	Sushi Domain Containing 4	−2.43	9.71E-07
Lin7a	Lin-7 Homolog A	−2.39	2.58E-05
Prrt1	Proline-Rich Transmembrane Protein 1	−2.20	5.78E-11
Rgs17	Regulator Of G-Protein Signaling 17	−2.11	1.49E-07
Gfap	Glial fibrillary acidic protein	−2.05	2.82E-07
Ssbp3	Single-Stranded DNA Binding Protein 3	−2.05	1.29E-09
Slc5a7	Solute Carrier Family 5 (Sodium/Choline Cotransporter), Member 7	−2.04	1.41E-07
Tubb4	Tubulin beta-4A chain	−2.00	8.33E-09
Gnai1	Guanine Nucleotide Binding Protein (G Protein), Alpha Inhibiting Activity Polypeptide 1	−1.99	3.74E-05
Gria1	Glutamate Receptor, Ionotropic, AMPA 1	−1.97	2.49E-05
Klf13	Kruppel-like factor 13	−1.96	3.37E-05
Mapt	Microtubule-Associated Protein Tau	−1.94	1.93E-06
Prkce	Protein Kinase C, Epsilon	−1.92	2.38E-06
Pick1	Protein Kinase C, Alpha-binding protein	1.90	9.65E-10
Glo1	Glyoxalase Domain Containing 1	1.97	1.11E-08
Sstr5	Somatostatin Receptor 5	1.99	2.30E-05

Fold Δ represents ENT1 null/wild-type ratio.

**Table 4:** A list of differentially expressed genes of the neurological disease functional pathway in the NAc between ENT1 wild-type and null mice.

ID	Gene Name	Fold Δ	*P* Value
Slc5a7	Solute Carrier Family 5 (Sodium/Choline Cotransporter), Member 7	−1.67	5.83E-05
Gpr116	Probable G-protein coupled receptor 116	−1.44	3.94E-05
Trerf1	Transcriptional Regulating Factor 1	−1.43	6.67E-05
Pik3r1	Phosphoinositide-3-Kinase, Regulatory Subunit 1	1.35	1.57E-05
Pdxk	Pyridoxal (Pyridoxine, Vitamin B6) Kinase	1.37	2.02E-04
Lphn3	Latrophilin-3	1.37	1.79E-04
Grm5	Glutamate Receptor, Metabotropic 5	1.38	2.22E-04
Spred2	Sprouty-Related, enabled/VASP homology 1 domainContaining 2	1.38	2.46E-04
Flrt2	Fibronectin Leucine Rich Transmembrane Protein 2	1.39	8.02E-05
Prkce	Protein Kinase C, Epsilon	1.41	1.59E-04
Vps13a	Vacuolar Protein Sorting 13 Homolog A	1.42	6.03E-05
Wwc1	WW And C2 Domain Containing 1	1.43	2.66E-04
Mcph1	Microcephalin 1	1.44	2.26E-04
Ppargc1a	Peroxisome proliferator-activated receptor gamma coactivator 1-alpha	1.48	9.90E-05
Scn2a	Sodium Channel, Voltage-Gated, Type 2, Alpha Subunit	1.49	1.73E-04
Kcnd2	Potassium Voltage-Gated Channel, Shal-Related Subfamily, Member 2	1.51	2.89E-04
Bbx	Bobby Sox Homolog	1.58	7.51E-05
Scn3a	Sodium Channel, Voltage-Gated, Type 3, Alpha Subunit	1.58	2.32E-04
Lin7a	Lin-7 Homolog A	1.65	6.68E-05
Ankrd11	Ankyrin Repeat Domain 11	1.65	2.36E-04
Atxn1	Ataxin 1	1.70	3.93E-05
Syne1	Spectrin Repeat Containing, Nuclear Envelope 1	1.70	5.77E-06
Cplx2	Complexin 2	1.92	3.31E-05

Fold Δ represents ENT1 null/wild-type ratio.

**Table 5:** A list of differentially expressed genes of the psychological disease functional pathway in the NAc between ENT1 wild-type and null mice.

ID	Gene Name	Fold Δ	*P* Value
Slc5a7	Solute carrier family 5 (choline transporter), member 7	−1.67	5.83E-05
Trerf1	Transcriptional regulating factor 1	−1.43	6.67E-05
Ptpre	Protein tyrosine phosphatase, receptor type, E	−1.32	7.27E-05
Prep	Prolyl endopeptidase	−1.30	4.22E-05
Chrnb2	Cholinergic receptor, nicotinic, beta polypeptide 2 (neuronal)	−1.27	2.88E-05
Eml4	Echinoderm microtubule-associated protein-like 4	1.28	2.16E-05
Rsrc2	Arginine/serine-rich coiled-coil 2	1.28	1.79E-05
Tle4	Transducin-like enhancer of split 4, homolog of Drosophila E(spl)	1.29	1.63E-04
Gnai2	Guanine nucleotide-binding protein (G protein), alpha inhibiting 2	1.31	2.80E-04
Zcchc17	Zinc finger, CCHC domain containing 17	1.31	2.38E-04
Pik3r1	Phosphatidylinositol 3-kinase, regulatory subunit, polypeptide 1 (p85 alpha)	1.35	1.57E-05
Grm5	Glutamate receptor, metabotropic 5	1.38	2.22E-04
Spred2	Sprouty-related, EVH1 domain containing 2	1.38	2.46E-04
Prkce	Protein kinase C, epsilon	1.41	1.59E-04
Ppargc1a	Peroxisome proliferative activated receptor, gamma, coactivator 1 alpha	1.48	9.90E-05
Scn2a1	Sodium channel, voltage-gated, type II, alpha 1	1.49	1.73E-04
Kcnd2	Potassium voltage-gated channel, Shal-related family, member 2	1.51	2.89E-04
Bbx	Bobby sox homolog (*Drosophila*)	1.58	7.51E-05
Scn3a	Sodium channel, voltage-gated, type III, alpha	1.58	2.32E-04
Lin7a	Lin-7 homolog A (*Caenorhabditis elegans*)	1.65	6.68E-05
Atxn1	Ataxin 1	1.70	3.93E-05
Syne1	Spectrin Repeat Containing, Nuclear Envelope 1	1.70	5.77E-06
Cplx2	Complexin 2	1.92	3.31E-05

Fold Δ represents ENT1 null/wild-type ratio.

### Reduced expression of GFAP in the striatum of ENT1 null mice by immunofluorescence

**Figure 2:**
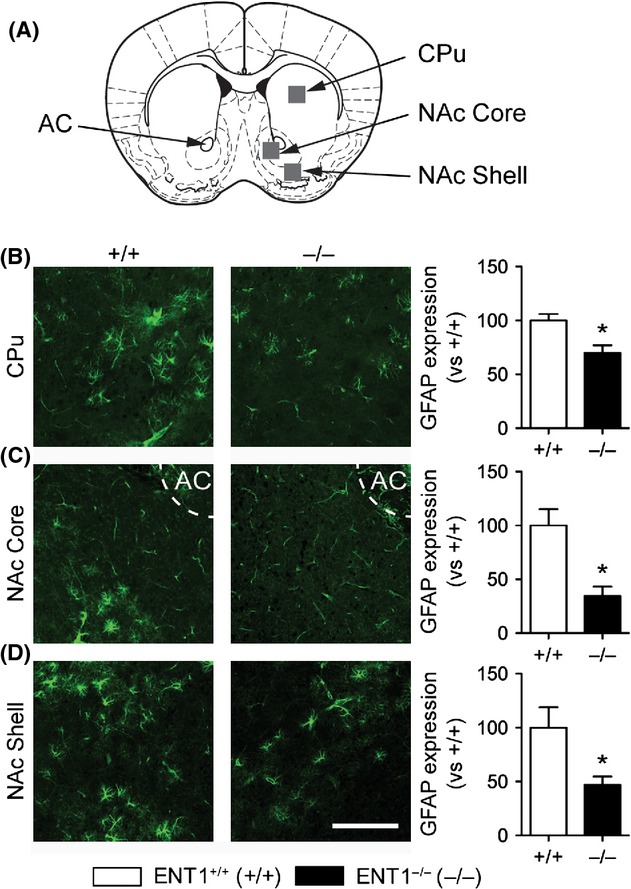
GFAP expression by immunohistochemistry is reduced in ENT1 null mice. (A) Schematic representation of the approximate location of the immunofluorescence image acquired from the caudate-putamen (CPu), the core region of the nucleus accumbens (NAc core), and the shell region of the NAc (NAc shell). Anterior commissure (AC) is highlighted as a landmark or the NAc core. Gray boxes are not to scale. Anatomic brain section was adapted from Paxinos and Franklin's stereotaxic atlas published in The Mouse Brain in Stereotaxic Coordinates, 3^rd^ edition, Copyright Elsvier (Paxinos and Franklin 2008). Reduced GFAP expression in the CPu (*n *=* *6 mice per genotype; (B), NAc core (*n *=* *5 mice per genotype; (C) and NAc shell (*n *=* *4–5 mice per genotype; (D) subregions of the striatum. Scale = 100 *μ*m. *P <* *0.05, compared to wild-type mice by unpaired two-tailed *t* test. All data are expressed as mean ± SEM.

Since GFAP was identified as a top candidate gene that was downregulated in the striatum of ENT1 null mice compared to wild-type mice, we investigated whether deletion of ENT1 was associated with reduced GFAP expression by immunofluorescence in the CPu, NAc core, and NAc shell (Fig.2A). We found that GFAP fluorescent expression was downregulated in the CPu [*t*_16_ = 3.42; *P *=* *0.004; Fig.2B] and NAc core [*t*_8_ = 3.74; *P *=* *0.006; Fig.2C] and NAc shell [*t*_7_ = 2.80; *P *=* *0.027; Fig.2D] of ENT1 null mice compared to wild-type littermates. Furthermore, immunofluorescence analysis revealed that GFAP was expressed mainly in the star-shaped astrocyte soma with some expression also observed in the end-feet (Fig.2B–D). Overall, ENT1 null mice showed reduced GFAP expression, which could impair the function of astrocytes in the striatum.

### GFAP promoter activity is downregulated in bitransgenic ENT1 null mice

**Figure 3:**
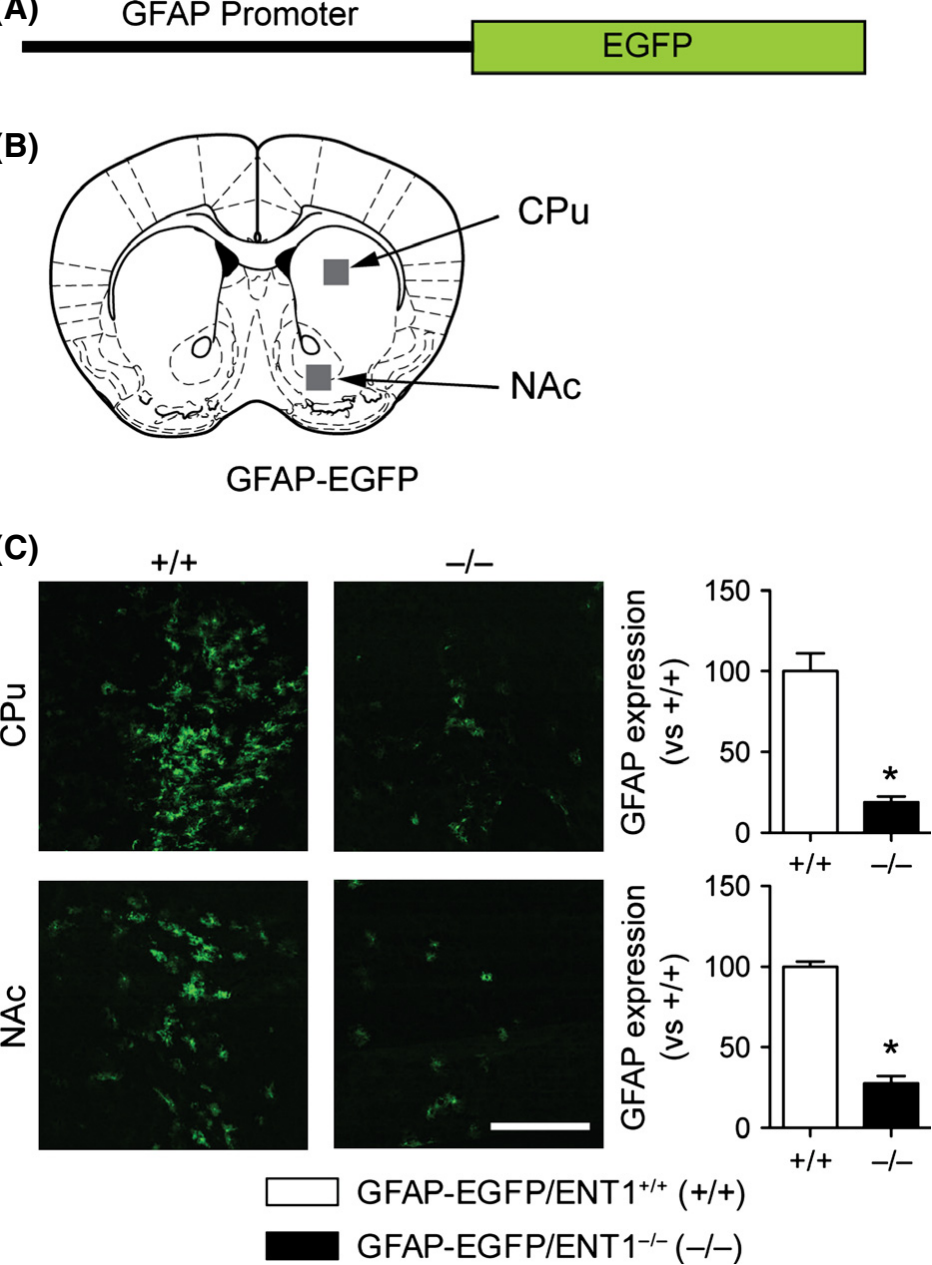
GFAP promoter activity was reduced in the striatum of ENT1 null mice expressing EGFP under the control of the GFAP promoter. (A) Schematic representation of the approximate location of the immunofluorescence image acquired from the caudate-putamen (CPu) and the nucleus accumbens. Gray boxes are not to scale. Anatomic brain section was adapted from Paxinos and Franklin's stereotaxic atlas published in The Mouse Brain in Stereotaxic Coordinates, 3rd edition, Copyright Elsvier (Paxinos and Franklin 2008). (B) Bitransgenic mice expressing EGFP under the control of the GFAP promoter (GFAP-EGFP) in ENT1 null background were generated to investigate GFAP promoter activity in ENT1 null mice. (C) Both striatal regions show a significant reduction in GFAP promoter activity in ENT1 null bitransgenic mice (*n *=* *4 per genotype per region). Scale bar = 100 *μ*m. *P <* *0.05, compared to GFAP-EGFP mice on a wild-type background by unpaired two-tailed *t* test. All data are expressed as mean ± SEM.

In order to investigate whether GFAP expression is altered at the promoter level, we utilized an EGFP reporter mouse under the control of the GFAP promoter (Fig.3A) on an ENT1 null or wild-type background in the CPu and NAc (Fig.3B). As shown in Figure3C, GFAP promoter activity was reduced by 81% and 72% in the CPu [*t*_6_ = 6.82; *P *<* *0.001] and NAc [*t*_6_ = 13.81; *P *<* *0.001], respectively in ENT1 bitransgenic mice. Therefore, this result suggests that deletion of ENT1 suppressed GFAP expression at the level of its promoter.

### Reduced expression of GFAP in the striatum of ENT1 null mice by real-time PCR and Western blot analysis

**Figure 4:**
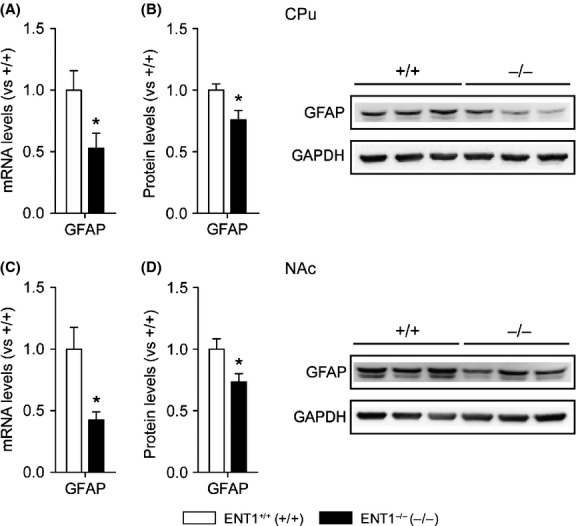
GFAP mRNA and protein expression is reduced in the CPu and NAc of ENT1 null mice. GFAP mRNA (*n *=* *11 mice per genotype; (A) and protein expression levels (*n *=* *5–8 mice per genotype; (B) were reduced in the CPu of ENT1 null mice. Similarly, GFAP mRNA (*n *=* *11–12 mice per genotype; (C) and protein expression levels (*n *=* *8–9 mice per genotype; (D) were reduced in the NAc of ENT1 null mice. GAPDH was used as a control. *P <* *0.05, compared to wild-type mice by Student's unpaired two-tailed *t* test. All data are expressed as mean ± SEM.

Next, we further functionally validated the decrease in GFAP expression in the striatum of ENT1 null mice compared to wild-type mice by real-time PCR and Western blot analysis. We found that GFAP mRNA expression was downregulated in both the CPu [*t*_19_ = 3.73; *P *=* *0.001; Fig.4A] and in the NAc [*t*_21_ = 3.40; *P *=* *0.003; Fig.4C]. Furthermore, Western blot analysis showed that GFAP protein levels were reduced in both the CPu [*t*_11_ = 2.76; *P *=* *0.019; Fig.4B] and NAc [*t*_15_ = 2.55; *P *=* *0.022; Fig.4D] of ENT1 null mice compared to wild-type mice.

### Inhibition of ENT1 downregulates astrocytic GFAP expression

**Figure 5:**
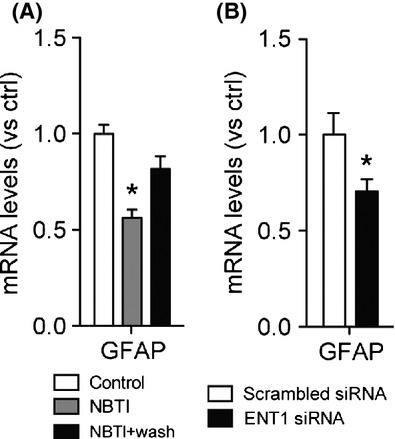
GFAP mRNA is reduced by pharmacological inhibition and siRNA knockdown of ENT1. (A) ENT1-specific inhibitor NBTI treatment for 24 h at 10 *μ*mol/L significantly reduced GFAP mRNA expression levels in a cerebellar (C8-D1A) astrocytic cell line (*n *=* *4 per condition). *P <* *0.05 compared to control group by one-way ANOVA followed Tukey post hoc test. (B) ENT1 siRNA treatment for 24 h significantly reduced GFAP mRNA expression levels in a cerebellar (C8-D1A) astrocytic cell line (*n *=* *6 per condition). *P < 0.05 compared to control group by unpaired two-tailed *t* test. The level of GFAP mRNA was determined by real-time RT-PCR using GAPDH as an internal normalization control. All data were expressed as mean ± SEM.

Previously, we showed that inhibition of ENT1 downregulates EAAT2 expression and function in astrocytes through adenosine A1 receptor signaling (Wu et al. 2010, 2011) and also reduces expression of AQP4, but not EAAT1 or GS expression (Lee et al. 2013). To further investigate whether ENT1 regulates GFAP, we used C8-D1A from the mouse cerebellum (Alliot and Pessac 1984). In Fig.5, ENT1 inhibition *via* 10 *μ*mol/L NBTI (nitrobenzylthioinosine) downregulated GFAP expression, which was restored following washout [*F*_2,9_ = 17.30; *P *<* *0.001; Fig.5A]. Furthermore, ENT1-specific siRNA treatment, which we have shown previously to reduce ENT1 mRNA levels (Lee et al. 2013), reduced GFAP mRNA expression in the C8-D1A cell line relative to a scrambled siRNA [*t*_10_ = 2.35; *P *=* *0.040; Fig.5B].

## Discussion

Our results show that GFAP was reduced in the striatum of ENT1 null mice compared to wild-type littermates using microarray and validation by RT-PCR, Western blot, and immunofluorescence. In addition, we found that this reduction in GFAP in ENT1 null mice was regulated at the level of the promoter as GFAP promoter-driven EGFP was also reduced in ENT1 null mice compared to wild-type littermates. We further confirmed this reduction in GFAP in astrocyte cell culture by inhibiting or knocking down ENT1. Overall, it appears that ENT1 plays an essential role in astrocyte function since GFAP plays a role in many of the functions of astrocytes and since we have previously observed that deletion, inhibition, or knockdown of ENT1 results in the reduction in EAAT2 and AQP4 (Wu et al. 2010, 2011; Lee et al. 2013).

Decreased expression of GFAP may reflect atrophy of astroglia, (Rajkowska et al. 2002; Fatemi et al. 2004), whereas increased GFAP expression is an indication of gliosis (Fatemi et al. 2004; Cho and Messing 2009; Middeldorp and Hol 2011). ENT1 null mice have been used to investigate ethanol use disorders and it is interesting that a number of studies have shown that GFAP immunoreactivity is increased during acute alcohol exposure, but long-term alcohol intake can reduce GFAP expression (Duvernoy et al. 1981; Franke et al. 1997; Crews et al. 2004; Miguel-Hidalgo 2005; Evrard et al. 2006). Thus, in ENT1 null mice, reduction in GFAP may cause atrophy of astrocytes and trigger downregulation of other astrocytic genes including EAAT2 and AQP4, which may mimic chronic alcohol phenotype. However, we found that expression of GS gene was similar between genotypes (Lee et al. 2013), suggesting possible upregulation of GS to compensate for a loss of GFAP-positive astrocytes in the striatum. Therefore, deficiency of ENT1 is specifically associated with a downregulation of EAAT2, AQP4, and GFAP. Further study is required to reveal causal relationship between reduction in GFAP and other genes.

Another mechanism that is possible for the reduction in specific astrocytic genes in ENT1 null mice is through DNA methylation since we have determined that the regulation of GFAP expression in ENT1 null mice is regulated at the promoter level. GFAP promoter activity not only regulates gene expression in general but also the expression pattern throughout the brain and the cell type specificity of its expression (Lee et al. 2008; Yeo et al. 2013). DNA methylation could be one mechanism that regulates GFAP expression as DNA methylation of the promoter region of GFAP has been shown to downregulate GFAP expression (Restrepo et al. 2011). During development, DNA methylation of the GFAP promoter plays a critical role in astrogliosis, neuronal development, and maturation of the CNS in general [reviewed in (Moore et al. 2013)]. DNA methylation of the GFAP promoter region has been shown to prevent STAT3 binding to reduce GFAP expression [reviewed in (Graff et al. 2011)]. We have previously shown that ENT1 null mice have reduced adenosine levels in striatal tissue lysate (Kim et al. 2011) and extracellular adenosine levels in the nucleus accumbens (Nam et al. 2011). The accumulation of intracellular adenosine concentration prevents the conversion of *S*-adenosylmethionine (SAM) to *S*-adenosylhomocysteine (SAH) essentially halting DNA methylation (Boison et al. 2013). The decrease in striatal tissue lysate adenosine levels in ENT1 null mice (Kim et al. 2011) suggests a decrease in intracellular adenosine levels, which would then be expected to increase global DNA methylation and in turn be responsible for the reduction in GFAP expression in ENT1 null mice. This would also suggest that the promoter regions of other genes could be regulated epigenetically through this adenosine-mediated shift in the transmethylation pathway. The CpG islands of the promoter region of EAAT2 have been extensively studied in their regulation of EAAT2 expression and it has been found that increased methylation decreases EAAT2 expression (Yang et al. 2010). Interestingly, EAAT2 is decreased in ENT1 null mice (Lee et al. 2013) and by ENT1 inhibition or downregulation in astrocytes (Wu et al. 2010, 2011) suggesting that an adenosine-mediated shift in the transmethylation pathway may play a role in the expression of EAAT2 as well. More research is required to characterize the potential epigenetic regulation of gene expression in response to ENT1-mediated dysregulation of adenosine.

Alexander's disease is a neurodegenerative genetic disease that has been shown to be a result of mutations in the GFAP gene. It is currently believed that the mutations in GFAP result in a gain-of-function and upregulation of GFAP rather than a loss of function of GFAP or downregulation of GFAP (Brenner et al. 2001). The gain of function of GFAP in models of Alexander's disease is suggested by the fact that mice null for GFAP develop normally (Pekny et al. 1995) and do not exhibit a phenotype that resembles Alexander's disease (Messing et al. 2012), while mice that overexpress GFAP die shortly after birth and have impaired cognition and spatial learning (Hagemann et al. 2013). Therefore, it is possible that using a pharmacological agent that dampens GFAP expression may be helpful in the treatment of Alexander's disease (Messing et al. 2010). It is interesting that in the present manuscript, we have demonstrated that ENT1 inhibition with a selective ENT1 inhibiter (NBTI) reduces GFAP mRNA levels in an astrocyte cell culture. However, further research is required to examine whether an ENT1 inhibitor reduces symptoms of Alexander's disease in animal models.

There is evidence that GFAP knockout mice are more susceptible to greater damage after brain injury (Nawashiro et al. 1998, 2000), blood–brain barrier disruption (Pekny et al. 1998) as well as infectious and inflammatory diseases (Liedtke et al. 1998). On the other hand, traumatic brain injury has been shown to upregulate GFAP and AQP4 resulting in dysregulated water flux across the plasma membrane and cellular edema suggesting that reducing GFAP expression may help to prevent some of the secondary damage that may result from brain injury (Marmarou et al. 2014). In addition, GFAP knockout mice have increased basal levels of GDNF that protect the medium spiny neurons of the striatum from metabolic and excitotoxic insults (Hanbury et al. 2003). Thus, the role of GFAP in precipitating brain injury or having a protective function remains to be further characterized.

It is of note that a decrease in GFAP expression was not detected in the NAc by microarray analysis although functional validation (immunofluorescence, promoter analysis, RT-PCR, and Western blot) shows that GFAP was clearly reduced in the NAc of ENT1 null mice. This indicates a limitation of microarray and the importance of functional validation. Interestingly, one of the most significantly downregulated genes observed from our microarray analysis was aquaporin 4 (AQP4; Table 2). We found that AQP4 was significantly reduced in the CPu of ENT1 null mice compared to wild-type mice. It is notable that AQP4 is the water channel of the CNS that is mainly expressed in astrocytes (Potokar et al. 2013). We previously identified reduced AQP4 in both the nucleus accumbens and caudate-putamen of ENT1 null mice compared to wild-type mice (Lee et al. 2013) and thus, our microarray experiment validates these findings.

In conclusion, impaired ENT1-dependent adenosine transporting activity results in a reduction in GFAP expression and astrocyte function in the CPu and NAc of mice. These data suggest that ENT1 may play a role in astrocyte function, which could contribute to some of the observed behavioral phenotypes of ENT1 null mice.

## Supporting Information

**Table S1**. Full list of differentially expressed genes in the CPu between ENT1 wild-type and null mice.

**Table S2**. Full list of differentially expressed genes in the NAc between ENT1 wild-type and null mice.
